# Supplementary material for: LOCALIZER: subcellular localization prediction of both plant and effector proteins in the plant cell
Source: Sci Rep. 2017 Mar 16;7:44598. doi: 10.1038/srep44598 (PMC5353544; doi:10.1038/srep44598)
Supplement: Supplementary File 1 [file srep44598-s1.pdf]

Supplementary Material

LOCALIZER: subcellular localization prediction of both plant and effector proteins in the plant cell

Jana Sperschneider[1,*], Ann-Maree Catanzariti[2], Kathleen DeBoer[3], Benjamin Petre[4], Donald M. Gardiner[5], Karam B Singh[1], Peter N. Dodds[6], Jennifer M. Taylor[6]

Table S1: The LOCALIZER predictor uses three classifiers for predicting chloroplast and mitochondrial transit peptides and NLSs. The protein net charge, isoelectric point and frequencies of amino acid classes (tiny, small, aliphatic, aromatic, nonpolar, polar, charged, basic, acidic) in the sequence were calculated using pepstats (Rice *et al.*, 2000). The grand average of hydropathicity (GRAVY), classes of predicted secondary structure elements (helix, turn, sheet) and aromaticity were calculated using ProtParam (Gasteiger *et al.*, 2005).

Classifier	Positive sequence set	Negative sequence set	Method of classification	Feature vector (58 features)
**Chloroplast**	Experimentally confirmed plant chloroplast/plastid transit peptides	N-termini of plant proteins with experimentally confirmed localizations nucleus, cytoplasm, mitochondria, secreted, membranes	Support vector machine	Protein net charge, isoelectric point, amino acid classes (9 features), amino acid frequencies (20 features), GRAVY, fraction of helix/turn/sheet (3 features), aromaticity, protein net charge of first 15 aas, isoelectric point of first 15 aas, amino acid frequencies of first 15 aas (20 features)
**Mitochondria**	Experimentally confirmed plant mitochondrial transit peptides	N-termini of plant proteins with experimentally confirmed localizations nucleus, cytoplasm, chloroplast, plastid, secreted, membranes	Support vector machine	
**Nucleus**	Collection of NLSs from experimentally confirmed eukaryotic nuclear proteins	-	Regular expression search and Hidden Markov Model search using NLStradamus	-

Table S2: Details of the subcellular localization prediction methods that were used for benchmarking. A version number of the software is given where provided by the authors.

Name	Localizations that can be predicted	Parameters	Version	Reference
**LOCALIZER**	Chloroplast, mitochondria, nucleus, none	Default	1.0	This work
**ChloroP**	Chloroplast, none	Default	1.1	Emanuelsson *et al.* (1999)
**TargetP**	Chloroplast, mitochondria, secreted, none	Plant, default	1.1	Emanuelsson *et al.* (2000)
**WoLF PSORT**	Chloroplast, cytosol, cytoskeleton, endoplasmic reticulum, extracellular, Golgi apparatus, lysosome, mitochondria, nuclear, peroxisome, plasma membrane, vacuolar membrane	Plant, default	0.2	Horton *et al.* (2007)
**BaCelLo**	Chloroplast, mitochondrion, secretory pathway, cytoplasm, nucleus	Plant, default	-	Pierleoni *et al.* (2006)
**YLoc**	chloroplast, nucleus, cytoplasm, mitochodrion, secretory pathway	YLoc-LowRes, plants	-	Briesemeister *et al.* (2010)
**YLoc (dual localization)**	nucleus, cytoplasm, mitochondrion, plasma membrane, extracellular space, endoplasmic reticulum, peroxisome, Golgi apparatus, vacuole, chloroplast	YLoc+, plants	-	
**Predotar**	Plastid, mitochondria, endoplasmic reticulum, none	Plant	1.03	Small *et al.* (2004)
**NLStradamus**	Nucleus, none	Default	1.8	Nguyen Ba *et al.* (2009)
**PredictNLS**	Nucleus, none	Default	1.3	Cokol *et al.* (2000)

Table S3: The benchmark set of eukaryotic effectors and effector candidates that have been studied in GFP localization experiments.

Data set	Reference	Number of proteins in set
**Subcellular localization of the *Hpa* RxLR effector repertoire**	Caillaud *et al.* (2012)	45
***Melampsora larici-populina* candidate effectors**	Petre *et al.* (2015b)<br>Petre *et al.* (2015a)	29
Puccinia striiformis* f. sp. *tritici candidate effectors	Petre *et al.* (2016)	16
**Literature search on effectors with localization data**	-	17

Table S4: Localization prediction results for rust effector candidates. Correct predictions are marked in bold.

Protein	Protein length (aas)	Experimental localization	LOCALIZER	ChloroP	TargetP	WoLF PSORT	YLoc	Predotar	BaCelLo
PGTG_00164	118	Chloroplast	Nucleus	-	Mitochondrion	Nucleus	**Chloroplast**	**Chloroplast**	**Chloroplast**
PGTG_06076	336	Chloroplast	**Chloroplast**	**Chloroplast**	**Chloroplast**	**Chloroplast**	**Chloroplast**	**Chloroplast**	**Chloroplast**
PGTG_13278	477	Nucleus	**Nucleus**	-	-	**Nucleus**	**Nucleus**	-	**Nucleus**
PGTG_15899	133	Nucleus	Mitochondrion, **Nucleus**	Chloroplast	Chloroplast	Chloroplast, Mitochondrion	Mitochondrion	-	Chloroplast

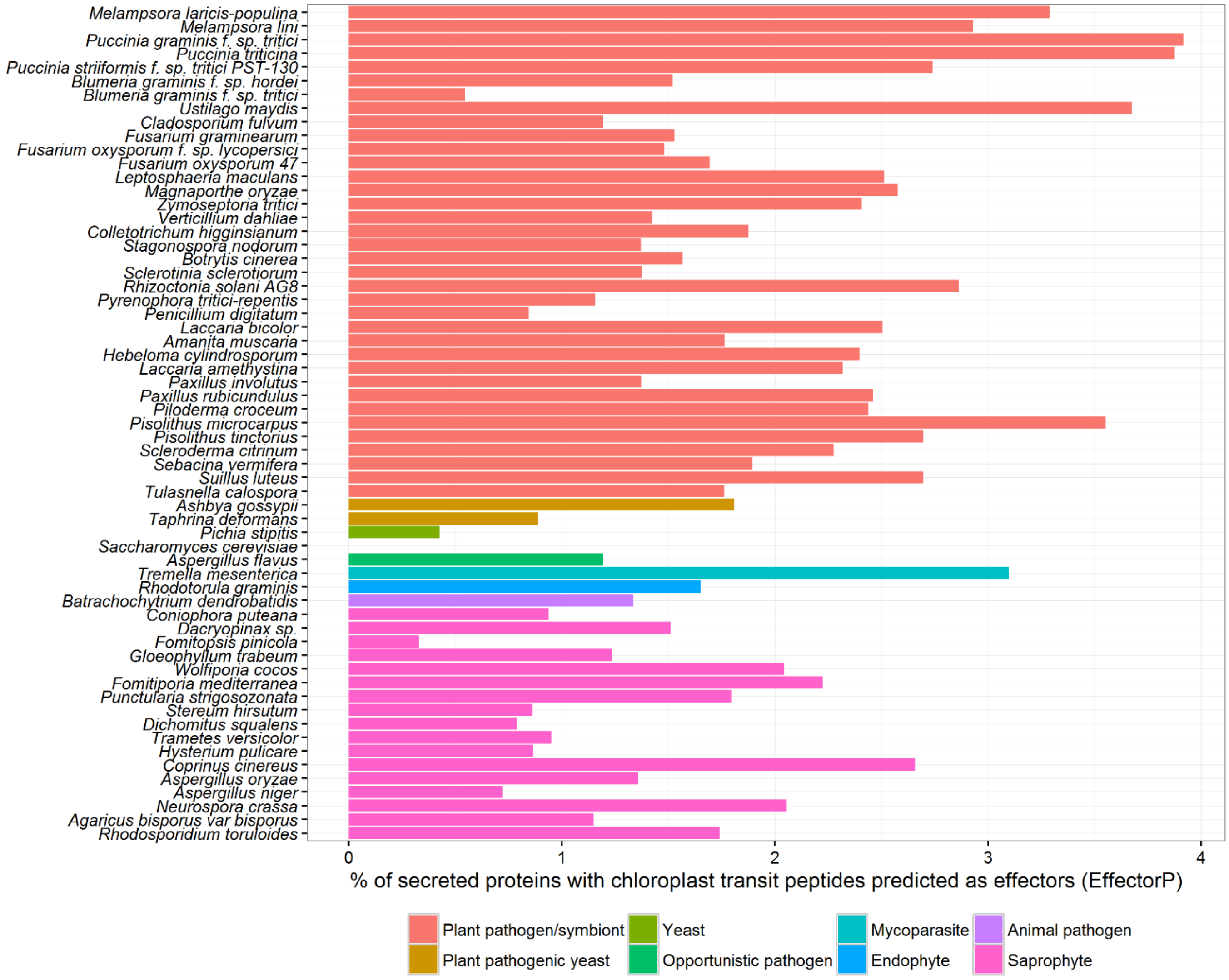

Fig. S1: The percentage of secreted proteins with predicted chloroplast transit peptides that are also predicted as effectors (EffectorP) is shown across fungal species.

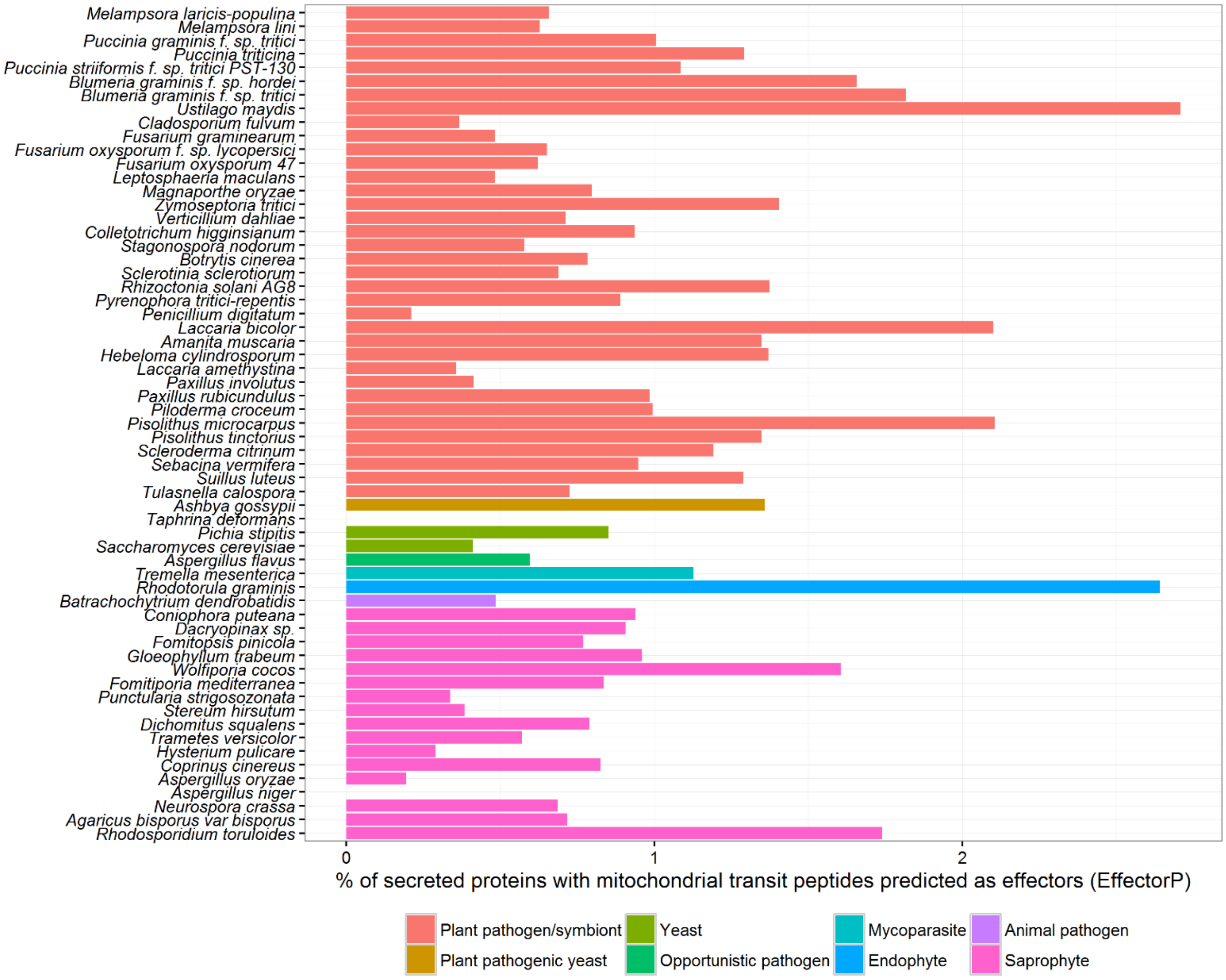

Fig. S2: The percentage of secreted proteins with predicted mitochondrial transit peptides that are also predicted as effectors (EffectorP) is shown across fungal species.

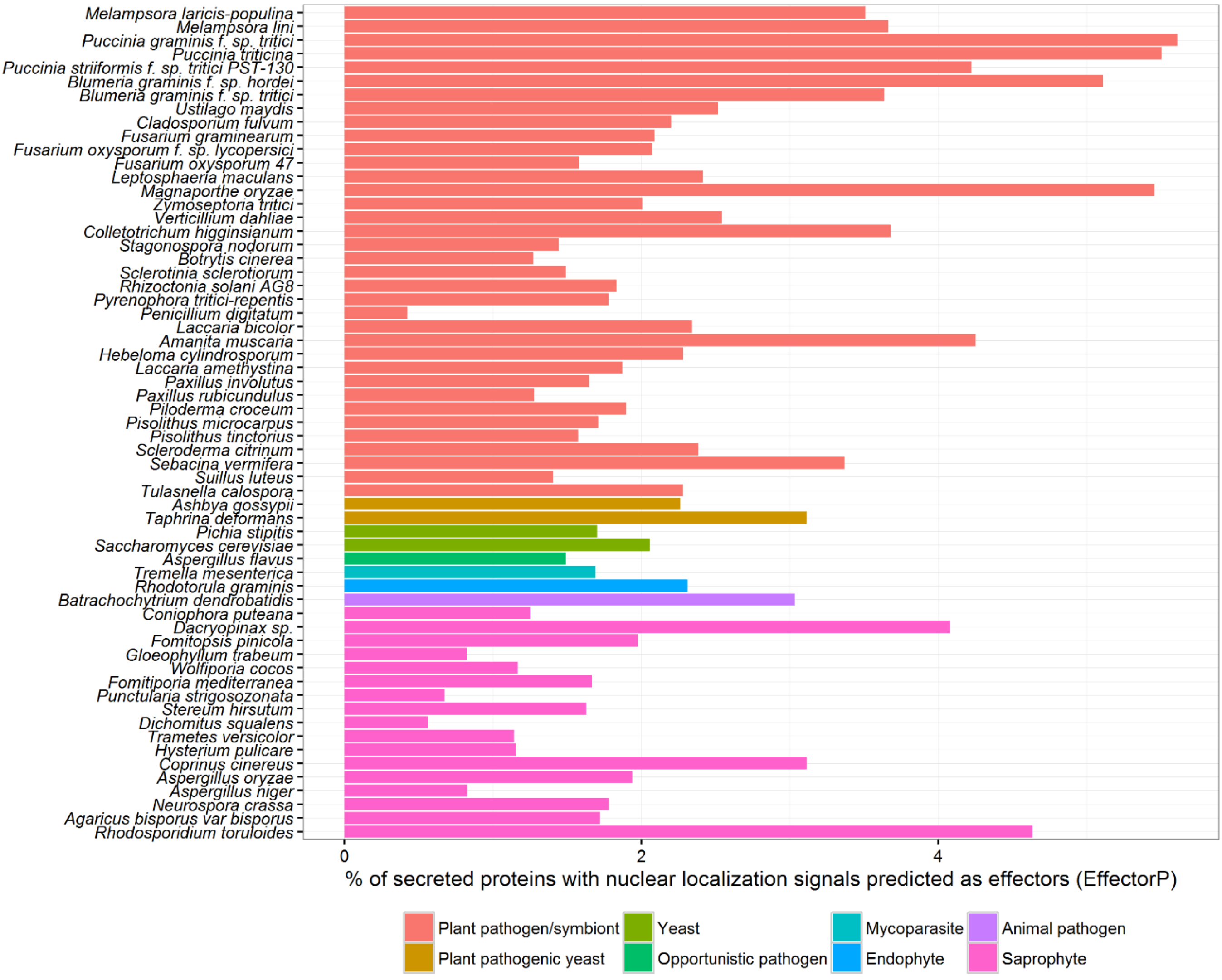

Fig. S3: The percentage of secreted proteins with predicted NLSs that are also predicted as effectors (EffectorP) is shown across fungal species.

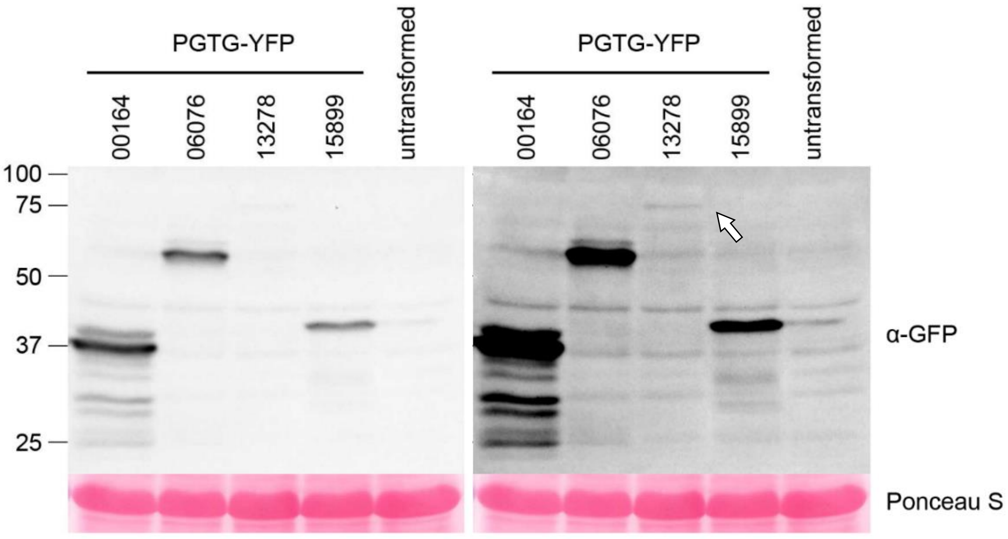

Fig. S4: Immunoblot analysis of YFP-tagged rust effector candidates. Protein extracts from *Nicotiana tabacum* leaf tissue (uninfiltrated) and leaf tissue transiently expressing *Puccinia graminis* f. sp. *tritici* (PGTG) effector candidates fused to yellow fluorescent protein (YFP) were analysed by immunoblotting with anti-GFP (α-GFP). Intact fusions were detected for all proteins, and only PGTG_00164 showed truncated products, which may correspond to cleaved YFP (~27 kDa). Image on the right shows a longer exposure time, which was required to visualise a protein band corresponding to PGTG_13278-YFP (indicated by arrow).

Predicted molecular weights are 40, 64, 81, and 40 kDa, respectively. Position and size (kDa) of protein molecular mass standards are indicated. Lower panel shows Ponceau S staining of protein bands on the blotted nitrocellulose membrane, indicating even protein loading.

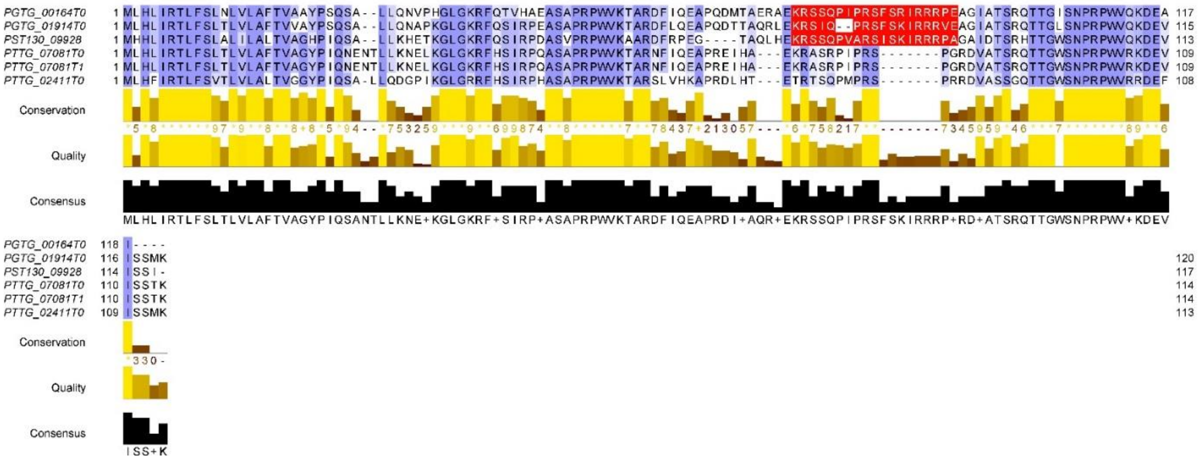

Fig. S5: Sequence alignment of PGTG_00164 and its rust homologs. The predicted NLS is marked in red.

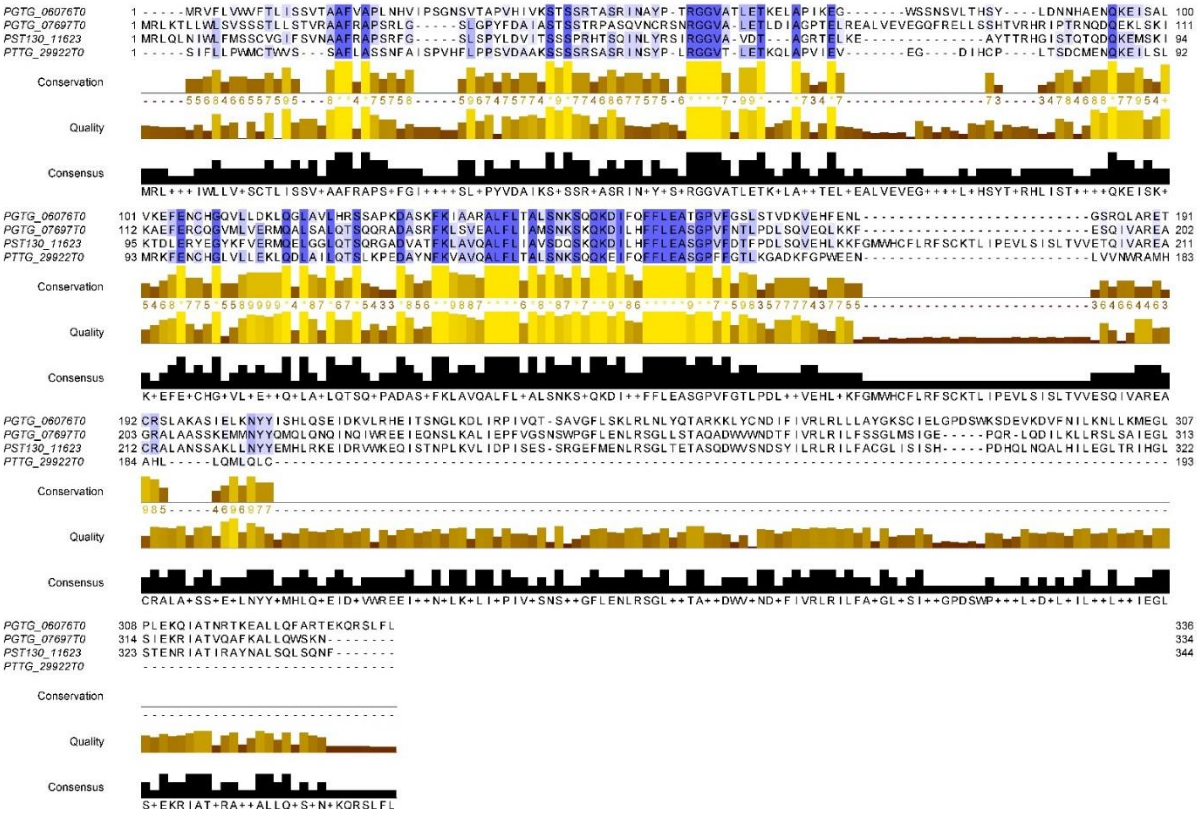

Fig. S6: Sequence alignment of PGTG_06076 and its rust homologs.

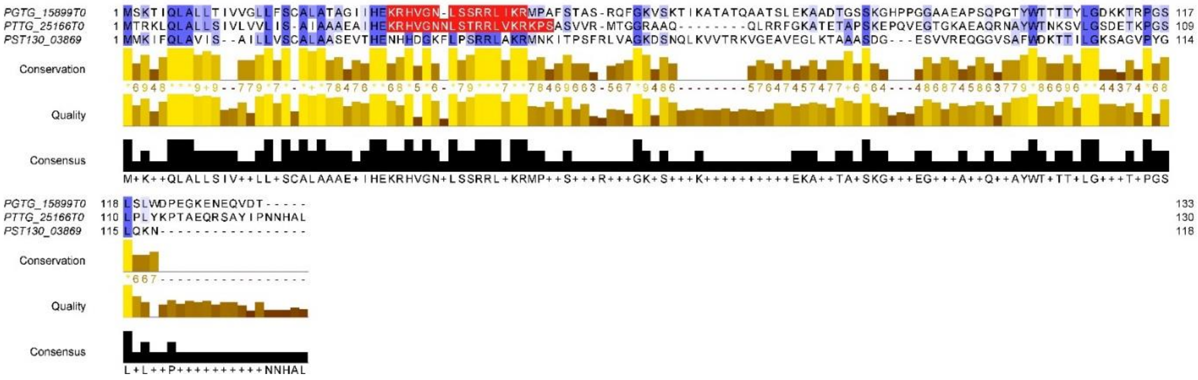

Fig. S7: Sequence alignment of PGTG_15899 and its rust homologs. The predicted NLS is marked in red.

Applying LOCALIZER to bacterial effectors targeting chloroplasts and mitochondria

We also tested LOCALIZER, which has been designed for plant proteins or eukaryotic effectors, on bacterial effectors that have been found to target chloroplasts or mitochondria. In plant mode, LOCALIZER scans for a transit peptide at the start of the protein only and we used plant mode to predict subcellular targeting of bacterial effectors. The enteropathogenic *Escherichia coli* effectors EspF and Map have been found to get imported into host mitochondria. For EspF, the mitochondrial targeting sequence is suggested to be in the N-terminal 24 aas, with the 16th leucine residue critical for host mitochondrial import (Nagai *et al.*, 2005). LOCALIZER in plant mode predicts a dual chloroplast/mitochondrial transit peptide in the first 26 aas. Interestingly, the mutation of L16 to E16 which abolishes mitochondrial import also leads to LOCALIZER dropping the mitochondrial prediction. For the Map effector, the N-terminal 44 residues are sufficient to target proteins to mitochondria (Papatheodorou *et al.*, 2006). However, LOCALIZER in plant mode predicts a chloroplast transit peptide in the first 27 aas. The *Anaplasma phagocytophilum* Ats-1 effector requires its N-terminus for mitochondrial localization and the cleavage site of Ats-1 is within N-terminal residues 45–60 (Niu *et al.*, 2010). LOCALIZER in plant mode predicts a mitochondrial transit peptide in the 21 aas. The *Pseudomonas syringae* type III effector HopG1 localizes to plant mitochondria but no transit peptide has been identified (Block *et al.*, 2010). For HopG1, LOCALIZER does not predict a transit peptide in plant mode. The *P. syringae* effectors AvrRps4 and HopK1 have been suggested to carry cleavable transit peptides that target them to chloroplasts (Li *et al.*, 2014), but AvrRps4 has also been reported in the cytosol and nucleus (Bhattacharjee *et al.*, 2011; Heidrich *et al.*, 2011; Sohn *et al.*, 2012). In plant mode, LOCALIZER does not predict transit peptides for AvrRps4 and HopK1. For the chloroplast-localized effectors HopO1-2 and HopR1 (de Torres Zabala *et al.*, 2015), LOCALIZER in plant mode predicts N-terminal chloroplast transit peptides.

Whilst LOCALIZER's predictions show agreement with experimentally determined bacterial effector localization, it is important to note that the amino acid composition of the N-terminal type III secretion signal of most *Pseudomonas syringae* effectors share a similar amino acid composition to chloroplast and mitochondrial transit peptides (Guttman *et al.*, 2002) and this similarity has been suggested to potentially resemble an evolutionary conserved mechanism shared by these organelles (Hicks & Galan, 2013). Therefore, the prediction of transit peptides in bacterial effectors might be due to the evolutionary history of bacterial secretion signals and large-scale bacterial genome screenings with LOCALIZER should be used with caution.

References

Bhattacharjee S, Halane MK, Kim SH, Gassmann W. 2011. Pathogen effectors target Arabidopsis EDS1 and alter its interactions with immune regulators. *Science* **334**(6061): 1405-1408.

Block A, Guo M, Li G, Elowsky C, Clemente TE, Alfano JR. 2010. The Pseudomonas syringae type III effector HopG1 targets mitochondria, alters plant development and suppresses plant innate immunity. *Cell Microbiol* **12**(3): 318-330.

de Torres Zabala M, Littlejohn G, Jayaraman S, Studholme D, Bailey T, Lawson T, Tillich M, Licht D, Bolter B, Delfino L, et al. 2015. Chloroplasts play a central role in plant defence and are targeted by pathogen effectors. *Nat Plants* **1**: 15074.

Gasteiger E, Hoogland C, Gattiker A, Duvaud S, Wilkins MR, Appel RD, Bairoch A 2005. Protein Identification and Analysis Tools on the ExPASy Server. In: Walker JM ed. *The Proteomics Protocols Handbook*: Humana Press 571-607

Guttman DS, Vinatzer BA, Sarkar SF, Ranall MV, Kettler G, Greenberg JT. 2002. A functional screen for the type III (Hrp) secretome of the plant pathogen Pseudomonas syringae. *Science* **295**(5560): 1722-1726.

Heidrich K, Wirthmueller L, Tasset C, Pouzet C, Deslandes L, Parker JE. 2011. Arabidopsis EDS1 connects pathogen effector recognition to cell compartment-specific immune responses. *Science* **334**(6061): 1401-1404.

Hicks SW, Galan JE. 2013. Exploitation of eukaryotic subcellular targeting mechanisms by bacterial effectors. *Nat Rev Microbiol* **11**(5): 316-326.

Li G, Froehlich JE, Elowsky C, Msanne J, Ostosh AC, Zhang C, Awada T, Alfano JR. 2014. Distinct Pseudomonas type-III effectors use a cleavable transit peptide to target chloroplasts. *Plant J* **77**(2): 310-321.

Niu H, Kozjak-Pavlovic V, Rudel T, Rikihisa Y. 2010. Anaplasma phagocytophilum Ats-1 is imported into host cell mitochondria and interferes with apoptosis induction. *PLoS Pathog* **6**(2): e1000774.

Papatheodorou P, Domanska G, Oxle M, Mathieu J, Selchow O, Kenny B, Rassow J. 2006. The enteropathogenic Escherichia coli (EPEC) Map effector is imported into the mitochondrial matrix by the TOM/Hsp70 system and alters organelle morphology. *Cell Microbiol* **8**(4): 677-689.

Rice P, Longden I, Bleasby A. 2000. EMBOSS: the European Molecular Biology Open Software Suite. *Trends Genet* **16**(6): 276-277.

Sohn KH, Hughes RK, Piquerez SJ, Jones JD, Banfield MJ. 2012. Distinct regions of the Pseudomonas syringae coiled-coil effector AvrRps4 are required for activation of immunity. *Proc Natl Acad Sci U S A* **109**(40): 16371-16376.
